# Treatment of Class III Malocclusion: Atypical Extraction Protocol

**DOI:** 10.1155/2017/4652685

**Published:** 2017-02-06

**Authors:** Fernando Pedrin Carvalho Ferreira, Maiara da Silva Goulart, Renata Rodrigues de Almeida-Pedrin, Ana Claudia de Castro Ferreira Conti, Maurício de Almeida Cardoso

**Affiliations:** ^1^Cora-Vilhena, Vilhena, RO, Brazil; ^2^Sagrado Coração University, Bauru, SP, Brazil; ^3^Department of Orthodontics, Sagrado Coração University, Bauru, SP, Brazil

## Abstract

The treatment of Angle Class III malocclusion is rather challenging, because the patient's growth pattern determines the success of long-term treatment. Early diagnosis and treatment are still highly discussed issues in orthodontic literature. This type of early intervention has been indicated more frequently in order to eliminate primary etiological factors and prevent an already present malocclusion from becoming severe. However, when a patient is diagnosed in adulthood, manipulation of the bone bases becomes extremely limited, as there is no longer any potential for growth. Treatments are restricted to dental compensations when possible or orthognathic surgery. However, owing to the high cost and inherent risk of the surgical procedure, this treatment option is often denied by the patient; in such a case, the orthodontist has little choice but to perform, where possible, compensatory treatments to restore a functional occlusion and improve facial esthetics. This article reports a case of Class III malocclusion in a patient who opted for compensatory treatment with lower molar extraction that allowed for correction of the midline and the overjet. Good facial esthetics and functional normal occlusion were achieved at the end of the treatment.

## 1. Introduction

Angle Class III malocclusion is the least common malocclusion. Its prevalence varies according to the surveyed area and is higher in Asian countries like Japan and Korea [1, 2]. Its prevalence in the general population in China is 15,69% [1] while that in Europe is only 2–6% [2]. It has a strong genetic component and is one of the most challenging malocclusions to treat.

When compared with normal occlusion, the lower posterior teeth occlude mesially in relation to the upper teeth, in Class III malocclusion cases. The anterior region also presents this discrepancy in the anteroposterior direction, seen as a reversal of the horizontal overlap of the incisors, with the incisal edges of the lower teeth located in front of those of the upper. The bone bases reflect a sagittal skeletal discrepancy between the maxilla and the mandible. Development of the malocclusion can include skeletal retrusion of the maxilla, skeletal protrusion of the mandible, or a combination of these two factors [3, 4]. According to Guyer et al. [5], in a study with 5- to 15-year-old Class III patients, 57% had maxillary retrusion, irrespective of whether or not they presented with mandibular prognathism. Studies on the multifactorial etiology of Class III malocclusions show that maxillary retrognathism is as common as mandibular prognathism [3, 5].

Individuals with Class III malocclusion may present, as standard features of growth, excessive cranial prominence, mid-facial deficiency, lower lip prominence, and mandibular body that is often rotated forward and upward [4, 6]. Those patients often face the possibility of undergoing orthosurgical treatment when craniofacial growth is finished, since the face tends to reveal an unfavorable growth pattern over time. The choice of treatment is even more limited and challenging when a late diagnosis is made. For some patients, orthognathic surgery is the best option. It is a corrective procedure for the skeletal discrepancy, and if favored when the bone deformity is severe and excessively affects the facial appearance of the patient [7]. However, in borderline cases, compensatory orthodontic treatment may be opted for, since the esthetic balancing of the face is not always the major motive for treatment.

Some authors recommended extraction, orthodontic protocol, as one of the most common ways to treat these cases. Traditionally, the extraction of four premolars is the most common choice. Others reported alternative extractions for the treatment of Class III malocclusion [8, 9]. According to De Oliveira Ruellas et al. [10], when the third molars are present, the extraction of the first molar might be a good option to solve the problems of anterior-inferior crowding and vertical growth, as well as to attain a Class I molar relationship. Other authors, such as Capelozza Filho et al. [11], have treated this malocclusion with bonding and orthodontic brackets with specific angles to achieve compensation whenever possible.

The purpose of this clinical report is to present the case of an adult male patient with Class III malocclusion, with no complaints regarding facial esthetics, treated by an atypical extraction protocol of the lower molars, in order to achieve a stable and functional occlusion as similar to a natural compensation as possible.

## 2. Materials and Methods

### 2.1. Diagnosis and Etiology

**Figure 1:**
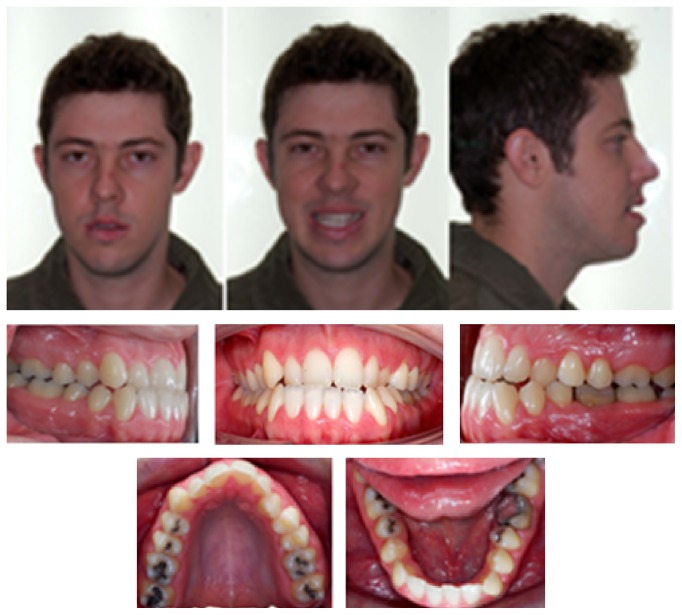
Pretreatment facial and intraoral photographs.

**Figure 2:**
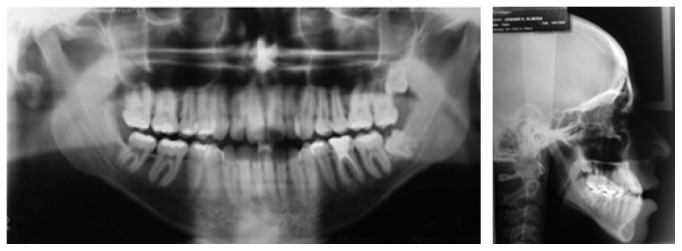
Pretreatment panoramic and cephalometric radiographs.

A male Caucasian patient sought orthodontic treatment for functional and esthetic complaints regarding his smile. Diagnostic tests were conducted to identify the problem and seek out possible treatment alternatives. Frontal facial analysis showed a decreased zygomatic projection, an increased vertical growth in the lower face, and an asymmetrical appearance (deviation to the right side), without lip sealing (Figure 1). In the lateral view, a concave profile was evident, with an increased chin-neck line, protrusion of the lower lip, and an inadequate zygomatic projection (Figure 1). The lateral face radiograph confirmed the findings of the facial analysis: a vertical growth pattern, protruded mandible, proclined upper incisors, and upright lower incisors, pointing towards compensation (Figure 2).

Cephalometric analysis showed a skeletal Class I malocclusion (ANB, 0.13°), with a well-positioned maxilla, a slight mandibular protrusion (SNA, 82.39°; SNB, 82.27°), and a hyperdivergent growth pattern (SN-MP, 44.17°). The angle between the upper incisors and the N-A line was 24.0°, and the angle between the latter and the lower incisors was 17.62°, verifying the lingual mandibular incisors. This position was confirmed by the reduction in the value of IMPA (73.96°). The interincisal angle was 138.1° (Table 1).

The panoramic radiograph showed the presence of all permanent teeth except 18 and 48 (Figure 2). The oral examination confirmed a Class III relationship of the molars and the canines that was more severe on the left side, with an inferior, midline deviation to the right, and an anterior cross-bite (Figure 1).

After radiological and facial evaluation, the patient was diagnosed with a Class III malocclusion, presenting a dolichofacial, asymmetrical, concave profile, with maxillary deficiency and a slightly increased mandibular growth. The etiology of skeletal Class III malocclusion in most cases is multifactorial, and therefore, the individuals affected by this anomaly demonstrate a combination of dental and skeletal factors [12, 13].

### 2.2. Therapeutic Options

Two different therapeutic approaches could have been followed for the treatment of the malocclusion: orthosurgical treatment or compensatory treatment. Orthognathic surgery was proposed for correction of the bone bases, but the patient refused relying on the lack of esthetic complains. Based on that, an orthodontic corrective treatment plan was indicated with the objective of dental compensation.

### 2.3. Objectives of the Treatment

The treatment aimed at (1) reestablishing a functional occlusion through dental compensation, (2) solving the sagittal imbalance, (3) correcting the midline deviation, and (4) improving the facial esthetics.

### 2.4. Treatment Progress

**Figure 3:**
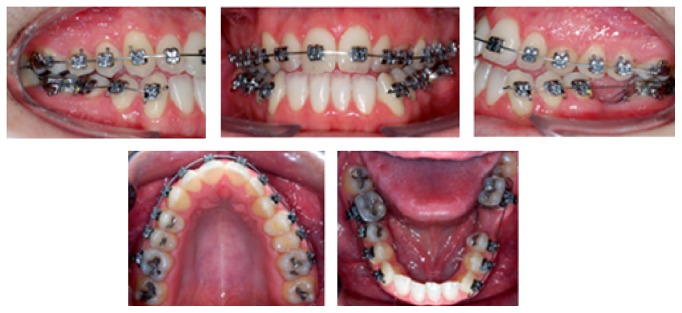
Intraoral views of treatment. Start of leveling, extraction of tooth 36, and segmented arch in the lower arch.

Fixed orthodontic treatment was initiated with self-ligating straight-wire brackets only in the upper arch. A decision to extract tooth 36 was made because of its destruction, which aided the treatment by correcting the inferior midline and reducing the dental mass, thus solving the anterior edge-to-edge bite (Figure 3).

**Figure 4:**
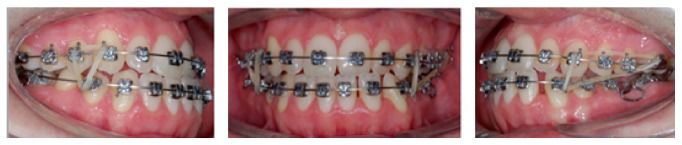
Intraoral views of treatment. Bonding the lower incisor, intermaxillary elastics, and loop to start closure extraction space.

**Figure 5:**
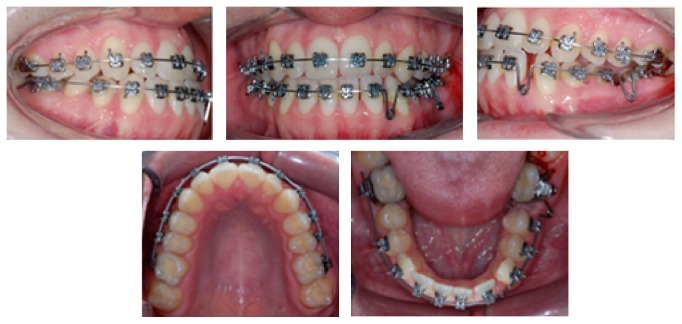
Intraoral views of treatment. End of leveling 0.017′′ × 0.025′′ stainless steel wire and retraction loops applied to close the extraction space and to correct the midline deviation.

**Figure 6:**
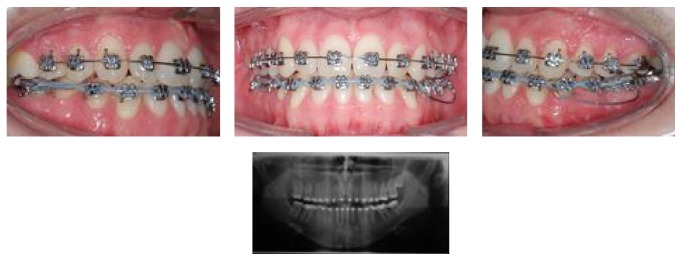
Intraoral views of treatment and panoramic radiograph. Bonding tooth 38, cantilever applied to upright tooth 37, and elastic chain to close the remaining spaces. Panoramic radiograph showing uprighted good position of tooth 38.

The wire sequence adopted for alignment and leveling was 0.014′′ NiTi, 0.016′′ NiTi, and 0.016′′ stainless steel wire. The lower brackets were also bonded on molars and premolars only at this point and after the installation of the 0.017′′ × 0.025′′ TMA wire, the lower anterior retraction was performed, only to the left side where tooth 36 was extracted (Figure 3). The use of Class III elastics was indicated at the same time, to facilitate the correction of overjet. In the upper arch, 0.016′′ × 0.022′′ NITI wire was used, followed by 0.018′′ and 0.020′′ bowflex steel arch to expand the left side for transverse adjustment. After achieving sufficient room for the incisors alignment, the lower anterior teeth were bonded (Figure 4). In order to close the extraction space and to correct the midline deviation retraction loops were applied (Figure 5). With the 0.019′′ × 0.025′′ stainless steel wire, an elastic chain was used for the mesialization of tooth 37. Tooth 38 was subsequently bonded, and the remaining spaces were closed with an elastic chain (Figure 6). The total treatment duration was 30 months.

## 3. Results

**Figure 7:**
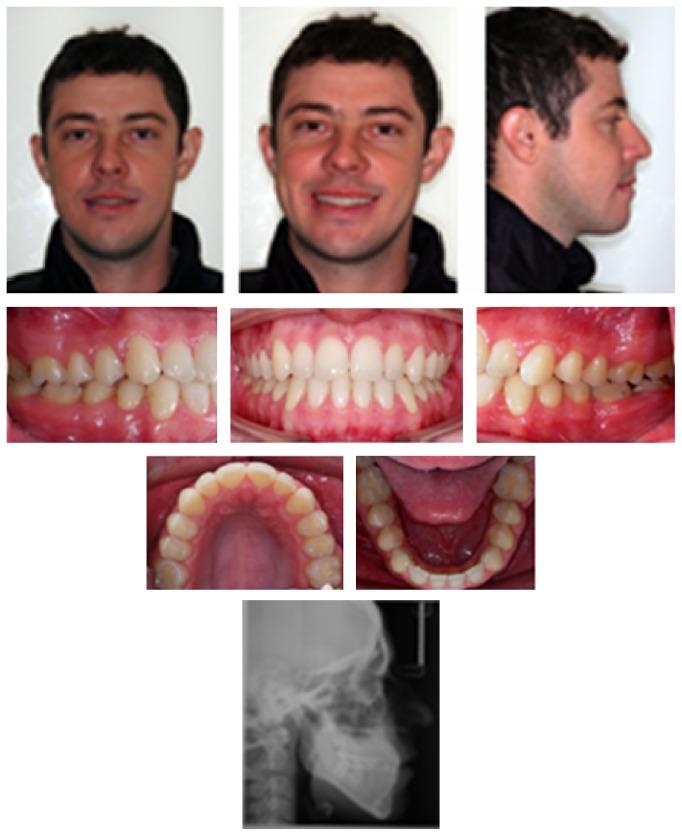
Posttreatment facial and intraoral photographs, and cephalometric radiograph.

At the end of treatment, a good occlusal relationship was achieved, with the correction of the overjet, coincidence of the midlines, and the correction of Angle Class III malocclusion, without the need for orthognathic surgery. It was also observed in the facial lateral view, passive lip sealing, and great improvement of facial esthetics (Figure 7).

**Figure 8:**
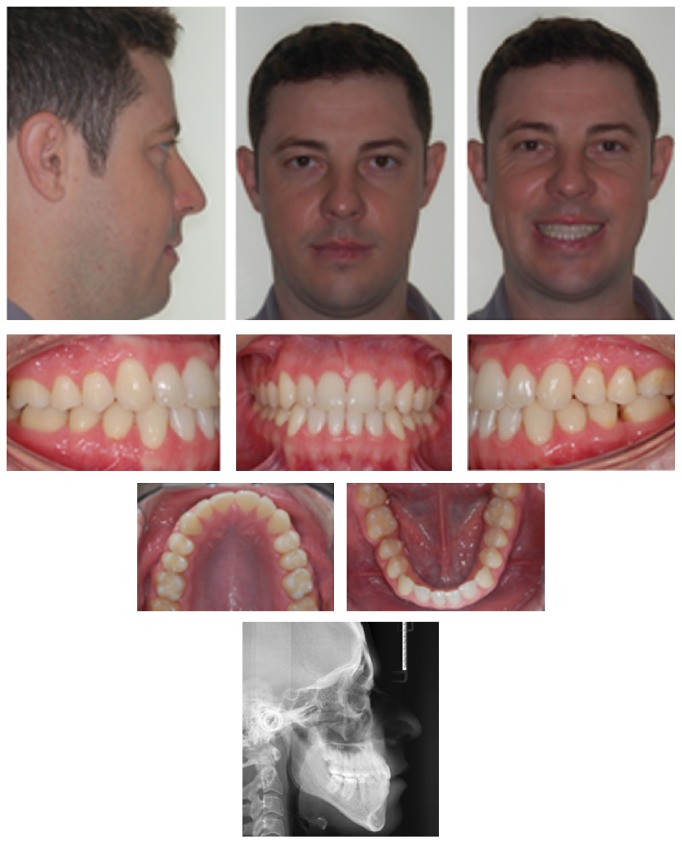
Posttreatment facial and intraoral photographs, and cephalometric radiograph (2 years after treatment completion).

New records were obtained 2 years after final treatment, last follow-up, and evaluation of the occlusion revealed Angle Class I molar relationship. The occlusion was stable and functional (Figure 8).

Final cephalometric analysis showed values for ANB, 0,69°; SNA, 82.53°; SNB, 81.84°; and a SN-MP, 43.42° defining the hyperdivergent growth pattern. It was observed an increase in the angle between the upper incisors and the N-A line from 24.0° to 27,14°, and for the lower incisors from 13,35° to 17.62°. A reduction in the value of IMPA from 73.96° to 68,1° was also noted. The interincisal angle was 138.8° (Table 1).

## 4. Discussion

Studies on the multifactorial etiology of Class III malocclusion show that maxillary retrognathism is as common as mandibular prognathism. Previous research has reported that 32–63% of the patients with skeletal Class III malocclusion have a maxillary deficiency or its combination with excessive mandibular growth [3, 5].

Most authors agree that an early intervention is the best option for Class III malocclusion treatment, because of the possibility of orthopedic management through facemask therapy, after maxillary expansion. This would redirect growth, making the malocclusion correction possible [14, 15].

Treatment options at later stages are limited, restricted to orthosurgical approach to correct bone discrepancies or orthodontic treatment aimed at correcting malocclusion through dental compensation. Frequently, the treatment plan includes extractions, and the use of intermaxillary elastics. This, however, has no impact on the facial esthetics, since the skeletal problem remains uncorrected [13, 16, 17]. Even considering this advantage, some authors still have reported success performing the compensatory treatment protocol [4, 16–20].

Despite being the most indicated treatment option in these cases, the inherent risk and high cost of the orthosurgical procedures make patients reluctant to accept it [6, 17, 21, 22].

In this case the patient also opted for orthodontic treatment without orthognathic surgery. In order to make possible the lower compensation and midline correction, extraction of tooth 36 was necessary. De Oliveira Ruellas et al. [10] and Sandler et al. [23] indicated the extraction of the first molars as a feasible treatment option in the presence of extensive caries, apical pathologies, significant restorations, severe crowding in the posterior region or anterior open bite. The option to extract the first molar depends on the presence and position of the third molar.

For the correction of anterior cross-bite and the normalization of the molar relationship, Lin and Gu [24] suggested the extraction of the second molar as the best option, as long as the patient had the third molar. This was in concurrence with a previous report by M. E. Richardson and A. Richardson [25], supporting the idea that the third molar can take the place of the second molar.

The contraindication for lower molar extraction is the difficulty in closing the space [10]. However, in the case described here, most of the space was used for the retraction of the anterior teeth, midline correction, and obtaining an adequate overjet.

The treatment options for orthodontic compensation in such patients include multiple extraction patterns. The extraction of the lower incisors is a good option for moderate Class III cases or edge-to-edge bite [26]. Some authors may suggest premolar extraction [10, 24]. The extraction of four premolars is not indicated in cases of severe malocclusion, or when the upper and lower teeth are well aligned, or when the lower crowding is not severe, since it can handicap the development of the jaw. The extraction of the third molars can be an alternative in these situations. However, the space created with the extraction of the third molars is limited, compared to that with the second molar extractions, which can be critical for the correction of the molar relationship and the anterior cross-bite [22].

A common strategy for orthodontic compensation with or without extraction is the use of intermaxillary Class III elastics, causing mesial movement of the upper teeth and distal movement of the lower teeth, with proclination of the upper teeth and retroclination of the lower teeth [9, 27, 28]. In our case, since the use of elastics was indicated for this patient, they were utilized as an adjunct to mechanics.

In this case the retraction in the lower arch was performed with the aid of a segmented arch with retraction loop [29] only on the left side to promote overjet and midline deviation correction. A better control of the force moment generated by the retraction loop caused an adequate space closure and a good occlusion. The segmented mechanics was also indicated for the lower arch in order to prevent a protrusion of the lower incisors, which in this Class III case was not recommended. Based on that, the incisors were bonded only when enough room for their alignment was provided.

Overall, the straight-wire mechanics associated with segmented arches in this case report achieved a good occlusion.

## 5. Conclusion

The Class III malocclusion was successfully treated by atypical extraction of only one lower molar. This less invasive approach was a feasible option for the patient who declined the orthosurgical alternative. The excellent esthetic and functional treatment outcome was possible, in large part, by the patient compliance.

## Figures and Tables

**Table 1:** Cephalometric analysis (USP-standard).

Measurement	Norm	Initial	Final	Control (2 years)
SNA	82.0°	82.39°	82.53°	83.53°
SNB	80.0°	82.27°	81.84°	84.13°
ANB	2.0°	0.13°	0.69°	0.60°
SN-MP	32.0°	44.17°	43.42°	42.05°
1.NA	22.0°	24.08°	27.14°	27.79°
1.NB	25.0°	17.62°	13.35°	14.78°
1.1	131.0°	138.17°	138.82°	138.02°
